# Chemical and Thermal Treatment for Drying Cassava Tubers: Optimization, Microstructure, and Dehydration Kinetics

**DOI:** 10.3390/life13122355

**Published:** 2023-12-16

**Authors:** Ellyas Alga Nainggolan, Jan Banout, Klara Urbanova

**Affiliations:** 1 Department of Sustainable Technologies, Faculty of Tropical AgriSciences, Czech University of Life Sciences Prague, Kamýcká 129, 16500 Prague, Czech Republic; 2 Department of Bioprocess Engineering, Faculty of Biotechnology, Institut Teknologi Del, Jl. Sisingamangaraja, Sitoluama, Laguboti, Toba 22381, North Sumatera, Indonesia

**Keywords:** cassava tubers, chemical treatment, dehydration kinetics, microstructure, optimization, thermal treatment

## Abstract

Perishable commodities like cassava necessitate effective postharvest preservation for various industrial applications. Hence, optimizing pretreatment processes and modeling drying kinetics hold paramount importance. This study aimed to optimize cassava pretreatment using the central composite design of a response surface methodology while also assessing microstructure and dehydration kinetics. Diverse chemical and thermal pretreatments were explored, encompassing sodium metabisulfite concentrations (0–4% *w*/*w*), citric acid concentrations (0–4% *w*/*w*), and blanching time (0–4 min). The four investigated responses were moisture content, whiteness index, activation energy (*E_a_*), and effective moisture diffusivity (*D_eff_*). Employing five established drying models, suitability was appraised after optimal pretreatment conditions were determined. The findings revealed that moisture content ranged from 5.82 to 9.42% db, whereas the whiteness index ranged from 87.16 to 94.23. *D_eff_* and *E_a_* ranged from 5.06 × 10^−9^ to 6.71 × 10^−9^ m^2^/s and 29.65–33.28 kJ/mol, respectively. The optimal pretreatment conditions for dried cassava were identified by optimizing the use of 1.31% citric acid, 1.03% sodium metabisulfite, and blanching time for 1.01 min. The microstructure indicated that particular chemical and thermal pretreatment configurations yielded particles in the shape of circular and elliptical granules. The logarithmic model provided the most accurate description of the dehydration kinetics, with the highest *R*^2^ value (0.9859) and the lowest *χ*^2^, *RSME*, and *SSE* values of 0.0351, 0.0015, and 0.0123, respectively.

## 1. Introduction

The postharvest processing of cassava tubers plays a vital role in determining the overall quality and safety of the commodity. This phase serves as an intermediary stage between the raw materials and the final products. Clearly, the objectives of the Millennium Development Goals for sustainability align with the imperative to ensure food safety and combat poverty. Postharvest processing plays a crucial role in extending the storage life of highly perishable commodities, including vegetables, fruits, and tubers such as cassava. The exploitation of cassava in downstream activities is crucial, since it provides chances for lucrative market endeavors [1].

Cassava (*Manihot esculenta*) is a tuberous plant belonging to the *Euphorbiaceae* family. The total global cassava production in the year 2020 amounted to 302 million tons, with Africa accounting for 64% of the production, Asia contributing 27%, the Americas providing 8.9%, and Oceania contributing 0.1% [2]. Cassava is widely used as a primary dietary source in tropical countries, making it a staple meal for a significant portion of the population. Its substantial potential in the food business has also been acknowledged by researchers [3]. The moisture level of freshly harvested cassava tubers generally varies from 60% to 72.60% [4,5,6]. The presence of elevated levels of moisture in tubers has a detrimental impact on their shelf life, transportation, and marketability, leading to substantial losses during the post-harvest period. The studies conducted by Riley et al. [7] and Srikanth [8] revealed that an elevated moisture content in yam tubers resulted in microbial spoilage, accelerated degradation, and triggered chemical and biological responses. The utilization of cassava tubers through various drying methods is crucial in order to effectively tackle these difficulties.

The drying process incorporates heat and mass transfer, which play a significant role in diminishing the moisture content of raw materials and modifying the physicochemical characteristics of the resulting dried product [9,10]. The process of reducing moisture in food involves the application of heat to facilitate the movement of moisture from the inside to the surface of the food material. This is subsequently followed by diffusion and evaporation processes [11]. According to Kerkhof and Coumans [12], the energy consumption attributed to drying procedures in industrialized nations ranges from 7% to 15%. The drying procedures employed in food production can consume significant amounts of energy and have a notable influence on the overall quality of the resulting dried food product [13]. The pivotal importance of mass and heat transfer properties in biomaterials is evident in the context of drying, which necessitates the implementation of pretreatment strategies to mitigate energy consumption in the drying process. Various chemical and physical treatments, including soaking in distilled water, blanching, chemical immersion, and salt immersion, have been employed on different tubers prior to the drying process [14,15,16,17].

Additional variables that impact the rate of drying encompass the speed at which air velocity, the air temperature, and the relative humidity. The factors mentioned in this context are connected to the effective moisture diffusivity and activation energy of a food material. Effective moisture diffusivity is a crucial transport property that depends on both the drying conditions and the characteristics of the material [18]. According to Rafiee et al. [19], effective moisture diffusivity must be taken into account when designing moisture transfer procedures for food product drying. Additionally, the concept of activation energy is employed to characterize the level of ease associated with moisture transfer in these food items. The activation energy necessary for commencing the drying process, as well as representing moisture diffusion throughout drying, can be estimated using the Arrhenius equation [20,21].

The establishment of kinetic models pertaining to the drying process of cassava tubers is of paramount importance in comprehending their characteristics and dynamics throughout the drying procedure. Mathematical models can be utilized to make predictions about the patterns of moisture transfer in biomaterials. Moreover, mathematical models play a crucial role in the design of drying systems and the analysis of heat and mass transfer events. A correlation exists between the parameters of a simulation model and the conditions of the drying process. It has been found that semi-theoretical models are good for describing how food items dry and for measuring how much moisture is removed during drying processes [11,22]. In addition, the utilization of a drying model offers advantages pertaining to the preservation of products and subsequent food processing [23]. In the study conducted by Cosme-De Vera et al. [24], it was found that the utilization of mathematical models in the context of drying may effectively capture and explain the variability observed in experimental data. Furthermore, the application of these models can lead to a reduction in experimental errors, optimization of drying processes, reduction in energy consumption, and ultimately, an enhancement of profitability [25,26,27,28,29].

The stability of food items can be improved through the implementation of drying techniques, as the reduction in moisture content has a significant impact on biological activity and the occurrence of physical and chemical alterations throughout the storage period [30]. The drying time has a significant impact on the amount of energy needed [11]. The length of drying can be influenced by various aspects, such as the initial treatment of the material, the temperature at which drying takes place, the features and size of the material, and the type of containers used for drying [11,22]. The optimization of the pretreatment entails the formulation of parameters and the selection of ideal factor combinations in order to minimize energy consumption during drying processes and attain the most favorable outcomes. Chemical concentration, soaking time, blanching duration, and blanching temperature are often employed factors in the chemical and thermal preparation of tubers [31,32,33,34]. Response Surface Methodology (RSM) is a statistical technique employed to optimize pre-drying treatments. RSM has been utilized as an optimization technique in the context of chemical and physical treatments given to cassava tubers [35,36,37,38].

Previous research has looked at how to improve chemical and physical pretreatment methods for cassava tubers. This study, on the other hand, uses response activation energy and effective moisture diffusivity as its main variables. Hence, there is a scarcity or absence of available knowledge pertaining to the optimization of pretreatment procedures utilizing chemical and thermal techniques, specifically in terms of their impact on moisture content, whiteness index, activation energy, and effective moisture diffusivity. The primary aims of this study encompass three key objectives. Firstly, to enhance the parameters involved in the pretreatment processes of cassava tubers. Secondly, to conduct a comprehensive analysis and evaluation of the microstructure of cassava flour obtained through pretreatment, utilizing both chemical and thermal methodologies. Lastly, to assess and determine the most suitable model for the drying kinetics of cassava tubers, considering the optimal conditions achieved through pretreatment.

## 2. Materials and Methods

### 2.1. Sample Preparation

Freshly harvested mature cassava tubers were purchased from local farmers at the Laguboti Market in the Laguboti District of the Toba Regency in North Sumatra Province, Indonesia. Malang 4 was the variety of cassava used in this study due to its cultivation development in the regency. Prior to the pretreatment and drying procedures, the cassava tubers were sorted according to their size and the extent of physical injury, washed with clean (clear, odorless, and purified) water, peeled, and sliced vertically across their dimensions to a thickness of approximately 5 mm using a knife. To minimize injury to the tubers, sample preparation occurred within 24 h of cassava tuber collection [39].

### 2.2. Pretreatment and Drying Experiments

Various pretreatments were then applied to the cassava slices. The moisture content of fresh cassava slices was measured to be 68.31% ± 0.37. The experimental design for pretreatment involved three independent variables: sodium metabisulfite concentrations (0–4% *w*/*w*), citric acid concentrations (0–4% *w*/*w*), and blanching times (0–4 min). A total of 20 experimental variations were generated from the three levels and three factors using central composite design (CCD) with Design Expert 13.0.5.0 software (Stat-Ease Inc., Minneapolis, MN, USA). Each run was repeated three times, and the mean values of the data collected from these repetitions were recorded. The chemical treatment consisted of soaking cassava slices in various concentrations of sodium metabisulfite and citric acid solutions for 20 min. In the meantime, the thermal treatment consisted of variable durations of 80 °C steam blanching.

The treated samples were drained and then dried in a thin layer using a forced convection drying machine (400 W Food Dehydrator, ATHOME collection, West Jakarta, Indonesia) operating at an air velocity of 2 m/s. The drying temperature was set at 70 °C, following the methodology of a prior study [16]. The sample’s weight was constantly checked every 30 min until it remained unchanged for three consecutive readings, indicating that the samples had reached equilibrium with the drying conditions. The cassava slices were then milled with a dry milling machine (HR 2115 Dry Mill Blender, PT. Philips Batam, Batam, Indonesia) and sieved using an 80-mesh sieve prior to being preserved at room temperature in plastic sample bags for subsequent analysis. Four responses were investigated, including moisture content, whiteness index, activation energy, and effective moisture diffusivity.

### 2.3. Moisture Content Analysis

The moisture content of the dried samples was assessed by measuring the beginning and final moisture content of the samples using an oven-drying technique in accordance with the Association of Official Analytical Chemists (AOAC) [40] guidelines. The percentage of moisture content was determined on a dry basis using Equation (1).

(1) $$MC\left(\%\right)=\frac{W_{t}\left(g\right)}{W_{i}\left(g\right)}\times 100\%$$

In this context, *MC* represents the percentage of moisture content, *W_t_* denotes the weight of the water in the sample at a specific time *t*, and *W_i_* signifies the weight of the sample after drying to a constant weight.

### 2.4. Color Measurement

The color measurements of the samples were performed in this investigation using a colorimeter (CS-10, Hangzhou Caipu Technology Co., Ltd., Hangzhou, China). The equipment underwent calibration by utilizing standard white and black reference tiles prior to conducting measurements. The parameters that were assessed encompassed L* (representing brightness), a* (representing redness, where positive values indicate red and negative values indicate green), and b* (representing yellowness, where positive values indicate yellow and negative values indicate blue). The integration of these values was performed using the whiteness index (*WI*) Formula (2), as established in the work conducted by [41], in order to assess the whiteness index of cassava flour.

(2) $$WI=100-\sqrt{a^{*2}+b^{*2}+{\left(100-L^{*}\right)}^{2}}$$

### 2.5. Determination of Effective Moisture Diffusivity

The effective moisture diffusivity of cassava slices is determined by applying Fick’s second law of diffusion to slab geometry, as indicated by Equation (3). Subsequently, Equation (4) was converted into logarithmic form to facilitate the estimation of the moisture diffusivity (*D_eff_*) value.

(3) $$MR=\frac{M_{i}-M_{e}}{M_{o}-M_{e}}=\frac{8}{\pi^{2}}\sum_{n-1}^{\infty }\frac{1}{{\left(2n+1\right)}^{2}}exp\left(\frac{-\left(2n+1\right)\times \pi^{2}\times D_{eff}\times t}{4H^{2}}\right)$$

(4) $$Ln\left(MR\right)=Ln\left(\frac{8}{\pi^{2}}\right)-\left(\frac{\pi^{2}\times D_{eff}\times t}{4H^{2}}\right)$$

where *MR* represents the dimensionless moisture ratio, *M_i_* is the moisture content at *i* time (% db), *M_e_* is the equilibrium moisture content (% db), *M_o_* is the initial moisture content (% db), *H* is half of the slices thickness (m), *n* is a positive integer, *D_eff_* represents the effective moisture diffusivity (m^2^/s), and ratio *t* is the time of drying (min).

Equation (4) gives the linear representation of Fick’s law. Consequently, a graph of *Ln* (*MR*) versus time produced a slope equal to $\frac{\pi^{2}\times D_{eff}}{4H^{2}}$, thereby determining the effective moisture diffusivity as described by [42,43].

### 2.6. Determination of Activation Energy

The following Equation (5) represents the calculation of the activation energy using the Arrhenius equation:

(5) $$D_{eff}=D_{o}exp\left(-\frac{E_{a}}{RT_{a}}\right)$$

where *E_a_* represents the activation energy (kJ/mol), *R* represents the universal gas constant (8.3143 J/mol/K), *T_a_* represents the absolute air temperature (K), and *D_o_* represents the pre-exponential factor from the Arrhenius equation (m^2^/s).

### 2.7. Microstructure Analysis

A scanning electron microscope (SEM) (JSM-6510LA, Jeol Ltd., Tokyo, Japan) was utilized to examine the morphology of both the untreated sample and the sample treated with chemical, thermal, and their combined methods. The objective of the microstructure analysis was to assess the impact of pretreatments on the particle morphology of cassava flour. The effects of pretreatment configurations on the response, specifically moisture content, can be ascertained by examining the morphological structure. The images in the scanning electron microscope instrument were captured with an applied voltage of 10 kV.

### 2.8. Statistical Analysis

Randomizing the experimental order completely prevented systematic errors in the statistical analysis of this study. Response analysis used a number of statistical tools, such as determination coefficient (*R*^2^), adjusted determination coefficient (adj *R*^2^), coefficient of variation (*CV*), lack of fit, and analysis of variance (ANOVA). Response surface methodology devised with central composite design using Design Expert version 13.0.5.0 (Stat-Ease Inc., Minneapolis, MN, USA) was used for modeling and optimizing the pretreatment process. The process of optimization consisted of endeavors to minimize all parameters, including citric acid concentration, sodium metabisulfite concentration, and blanching time.

### 2.9. Dehydration Kinetics

Once the optimal pretreatment conditions were determined, drying experiments were carried out to determine the actual drying behavior of cassava slices. The moisture ratio (*MR*) was determined by utilizing the simplified Fick’s diffusion law, as expressed in Equation (3). The collected data were then statistically analyzed utilizing five commonly used thin-layer drying models, as presented in Table 1.

Using Minitab^®^ 19.1 (Minitab LLC, Pennsylvania, PA, USA) software, experimental data obtained for the optimized moisture ratio were subjected to nonlinear regression analysis using the selected drying model parameters in order to evaluate the model parameters. The sufficiency of the data fit was examined by analyzing four statistical parameters: root mean square error (*RMSE*), coefficient of determination (*R*^2^), sum of square error (*SSE*), and reduced chi-square (*χ*^2^). These parameters are represented by Equations (6)–(9).

(6) $$R^{2}=\frac{1-[\sum_{i=1}^{n}{\left({MR}_{pre,i}-{MR}_{exp,i}\right)}^{2}]}{[\sum_{i=1}^{n}{\left({MR}_{pre,i}-{MR}_{exp,i}\right)}^{2}]}$$

(7) $$SSE=\frac{\sum_{i=1}^{n}{\left({MR}_{pre,i}-{MR}_{exp,i}\right)}^{2}}{N}$$

(8) $$\chi^{2}=\frac{\sum_{i=1}^{n}{\left({MR}_{pre,i}-{MR}_{exp,i}\right)}^{2}}{N-z}$$

(9) $$RMSE={\left[\frac{\sum_{i=1}^{n}{\left({MR}_{pre,i}-{MR}_{exp,i}\right)}^{2}}{N}\right]}^{\frac{1}{2}}$$

where *N* represents the quantity of experimental units, *MR_pred,i_* represents the predicted values, *MR_exp,i_* represents the experimental moisture ratio values, *MR* represents the predicted moisture ratio, and *z* represents the drying model’s constant values. Higher *R^2^* values, lower *SSE*, lower *χ*^2^ values, and lower *RMSE* values were used to evaluate the quality of fit [49,50,51].

## 3. Results and Discussion

### 3.1. Pretreatment Parameters of Dried Cassava

The design configuration and experimental response data (moisture content, whiteness index, activation energy, and effective moisture diffusivity) are presented in Table 2. The three factors included in the experimental design are the concentration of citric acid, sodium metabisulfite, and the duration of blanching.

#### 3.1.1. Moisture Content

**Figure 1:**
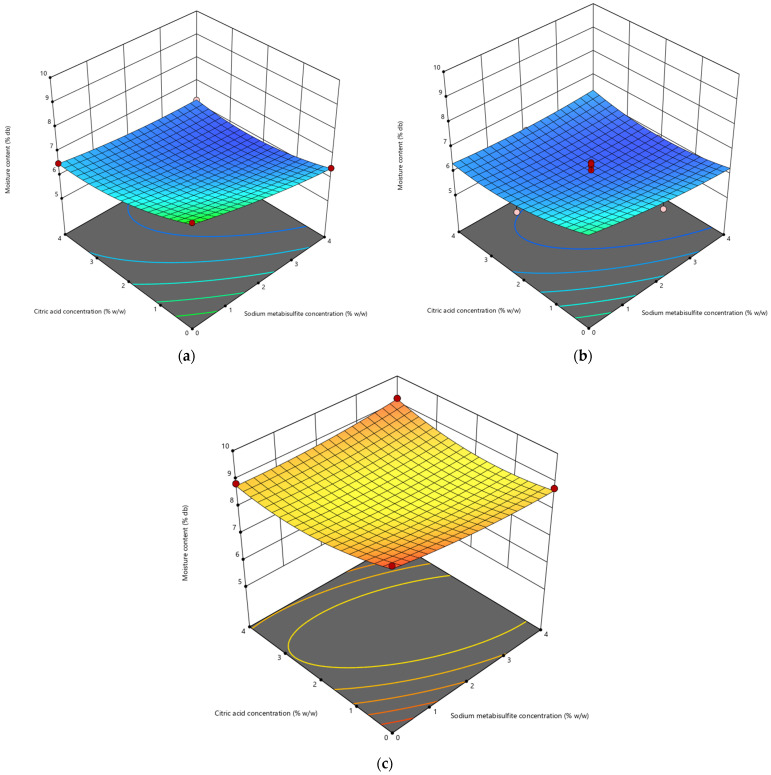
Impact of sodium metabisulfite concentration (X1) and citric acid concentration (X2) on the moisture content (Y) of cassava slices blanched for (**a**) 0 min, (**b**) 2 min, (**c**) 4 min.

Table 2 displays the percentage of moisture content in both untreated and treated cassava flour. In the presence of chemical pretreatment, the moisture content typically decreases, ranging from 5.82% to 9.98%. Figure 1 provides additional detail regarding the impact of chemical and thermal pretreatment on the percentage of moisture content. The findings of plot 1a indicate that an elevated concentration of chemical pretreatment has a discernible impact on the reduction in moisture content in the absence of blanching pretreatment. Increasing the blanching time (2 min) and sodium metabisulfite concentration (4% *w*/*w*) leads to better results in lowering the moisture content of dried cassava, as shown in Figure 1b. Previous studies reported comparable outcomes in yam samples subjected to sulfiting as a pretreatment [9,52]. In contrast, it was observed that the samples subjected to a blanching duration of 4 min exhibited the greatest levels of moisture content, which varied between 8.22% and 9.18%. This is due to the fact that blanched samples have higher values for water-binding capacity (WBC) than unblanched samples [53]. WBC values are predominantly influenced by the extent of starch fragmentation and gelatinization in the food product [54].

The utilization of citric acid and sodium metabisulfite in the chemical treatment of cassava tubers affects the moisture content of the dried cassava tubers. Table 2 demonstrates that the cassava tubers treated with citric acid have a higher moisture content compared to the tubers treated with sodium metabisulfite. This is consistent with the findings reported by Ngoma et al. [31], who discovered that sweet potatoes that were pretreated with citric acid had a higher moisture content compared to those pretreated with sodium metabisulfite. This related to the ability of sodium metabisulfite to enhance the removal of water from tuber slices by causing modifications in the permeability of cellular membranes [55].

#### 3.1.2. Whiteness Index

**Figure 2:**
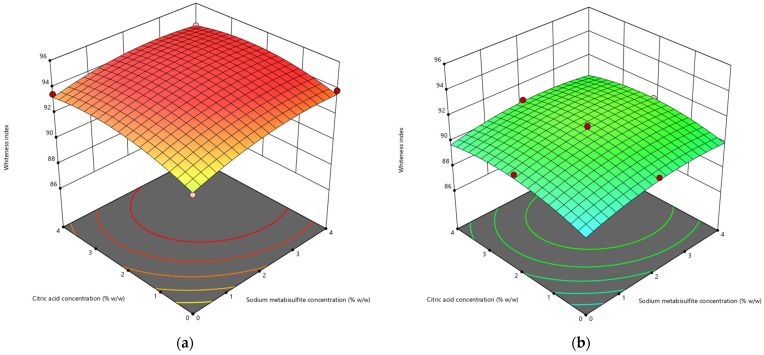

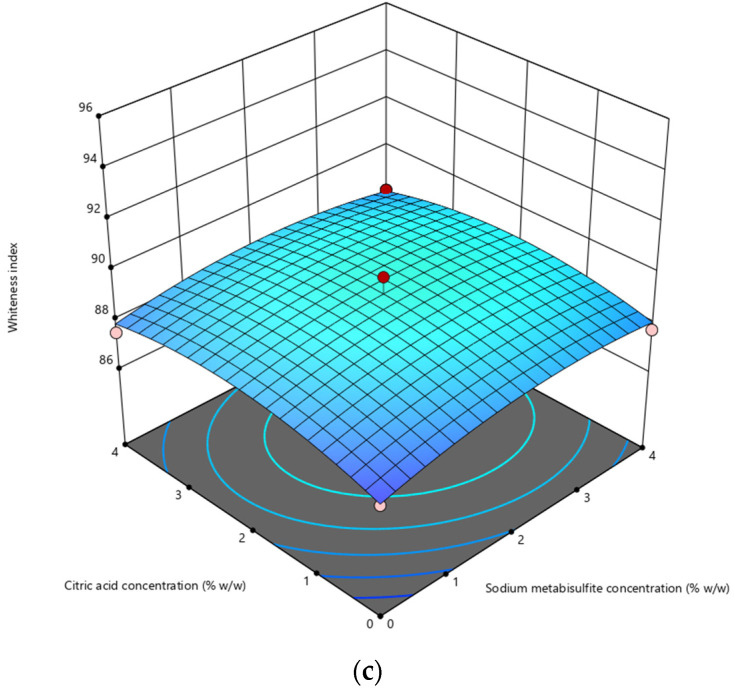
Impact of sodium metabisulfite concentration (X1) and citric acid concentration (X2) on the whiteness index (Y) of cassava slices blanched for (**a**) 0 min, (**b**) 2 min, (**c**) 4 min.

The role of color in flour-based food products is of great significance, as it significantly influences customer preferences for food quality. According to Anyasi et al. [56], the whiteness index is a measure that indicates the degree of whiteness in food items and the extent to which their color changes during the process of food preparation. Figure 2 presents a 3D surface plot that visually represents the influence of chemical and thermal preparation on the whiteness index. According to the findings presented in Table 2, the samples subjected to a 4 min blanching procedure without any chemical treatment exhibit the lowest whiteness index value. A study by Quayson et al. [57] found that the amount of color change caused by non-enzymatic processes is related to how long the blanching process lasts.

Conversely, the samples subjected to a chemical treatment including 4% citric acid and 4% sodium metabisulfite without undergoing blanching have the highest whiteness index value. Sodium metabisulfite and citric acid demonstrate notable impacts in the processing of cassava. According to a study conducted by Yongjie and Meiping [58], it was found that sulfites possess the ability to hinder both enzymatic and non-enzymatic processes. This characteristic renders them highly effective as color preservatives, especially when used in relation to perishable goods. Moreover, the utilization of metabisulfite offers supplementary advantages, such as its capacity to augment the nutritional composition of food items [59,60,61]. According to Ekeledo et al. [62], citric acid and similar preservatives have the ability to impede enzymatic reactions by means of oxygen removal throughout the course of food processing and storage. Application of citric acid resulted in an elevation of the whiteness index values in yam starches derived from four distinct cultivars [63].

#### 3.1.3. Effective Moisture Diffusivity

**Figure 3:**
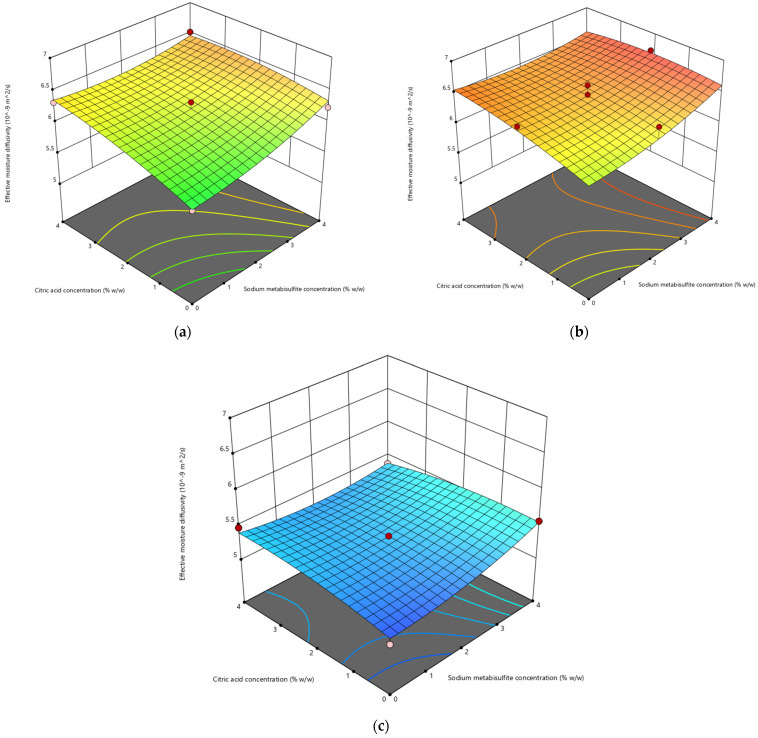
Impact of sodium metabisulfite concentration (X1) and citric acid concentration (X2) on the effective moisture diffusivity (Y) of cassava slices blanched for (**a**) 0 min, (**b**) 2 min, (**c**) 4 min.

Table 2 displays the values of effective moisture diffusivity for different pretreatment conditions. The *D_eff_* values obtained for the pretreated samples exhibit a range spanning from 5.06 × 10^−9^ to 6.71 × 10^−9^ m^2^/s. The findings of the study demonstrate that the application of both chemical and thermal pretreatment methods has had a significant impact on the diffusivity of moisture. Figure 3 shows a 3D surface plot that visually represents the influence of chemical and thermal pretreatment on effective moisture diffusivity. Additionally, it was discovered that subjecting the sample to a 2 min blanching pretreatment resulted in an increase in moisture diffusivity. Previous studies have also observed comparable outcomes in terms of the impact of pretreatment on moisture diffusivity during the drying process for blanched cassava slices [64].

It has been seen that sulfating as a pretreatment improves the process of moving water from the inside of cassava slices to the outside layers. The study conducted by Sahoo et al. [52] demonstrates that the processes of blanching and sulfiting have a substantial impact on the decrease in moisture content observed in yam slices. According to Ajala et al. [65] and Waramit et al. [66], the *D_eff_* values of cassava tubers ranged from 10^−11^ to 10^−7^ m^2^/s.

#### 3.1.4. Activation Energy

**Figure 4:**
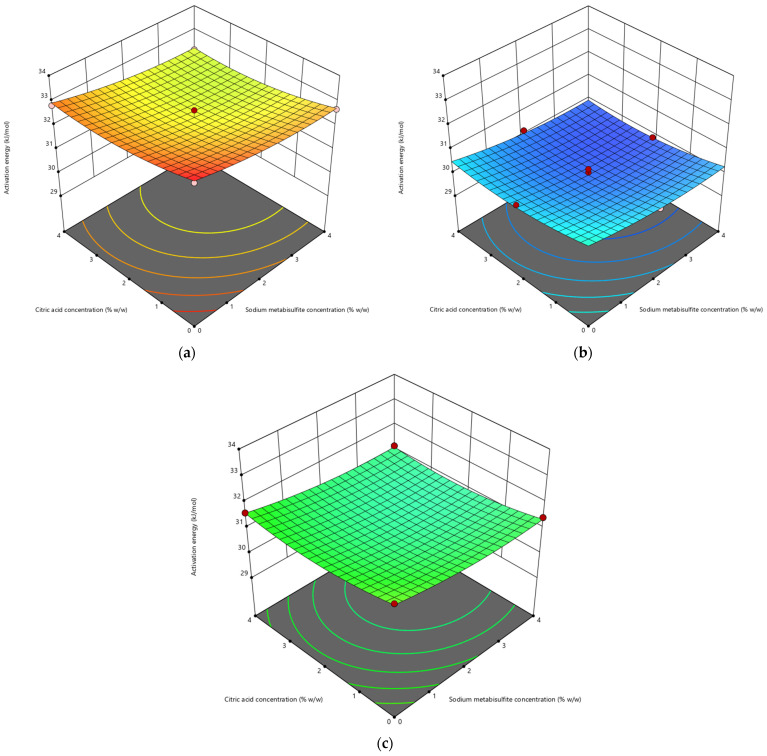
Impact of sodium metabisulfite concentration (X1) and citric acid concentration (X2) on the activation energy (Y) of cassava slices blanched for (**a**) 0 min, (**b**) 2 min, (**c**) 4 min.

The importance of activation energy in the drying process is essential, as it represents the energy required by drying equipment to facilitate the diffusion of moisture from the core to the outer surface of the product [67]. According to Menshutina et al. [68], activation energy also functions as an index for estimating internal changes in the behavior of biological materials during drying. The study yielded activation energy values ranging from 29.65 to 33.28 kJ/mol, as shown in Table 2. Pretreatment of samples generally results in the manifestation of reduced activation energy values. This suggests that the energy demand of the drying equipment for efficient drying is reduced. Figure 4 depicts the influence of pretreatment on the activation energy of thin-layer dried cassava. Upon comparing Figure 4a–c, it becomes evident that samples exposed to shorter blanching durations exhibit a tendency towards lower activation energy levels. The application of a brief 2-min blanching treatment has been shown to yield a considerable reduction in activation energy. Similarly, the utilization of chemical treatments including sodium metabisulfite and citric acid has also been found to result in a decrease in activation energy.

Ajala et al. [65] reported activation energies of 30.30 kJ/mol for cassava slices, 25.18–32.46 kJ/mol for untreated and treated yam slices [69], 28.576 kJ/mol for tapioca [70], and 22.70 kJ/mol for potato slices [18]. The variability in activation energy levels seen in tubers can be attributed to various factors, including but not limited to the tuber variety, stage of maturation, size of the sample, pretreatment methods, operating conditions, and the structural composition of the tissue [52]. However, the activation energy levels observed in this investigation are consistent with the recognized range for food materials, which spans from 12.7 to 110 kJ/mol [71].

### 3.2. Modeling and Optimization of the Pretreatment Parameters

Table 3 displays a multiple regression analysis and analysis of variance conducted on the experimental data and the model that was generated. The associations between the dependent and independent variables were determined by using a three-level factorial quadratic regression model based on the experimental data. The ANOVA demonstrates a statistically significant regression model (*p* < 0.05), as indicated by the small *p* values associated with the dependent variables. The results indicate that the concentration of sodium metabisulfite, citric acid concentration, and blanching time had a significant impact (*p* < 0.05) on all tested responses.

**Figure 5:**
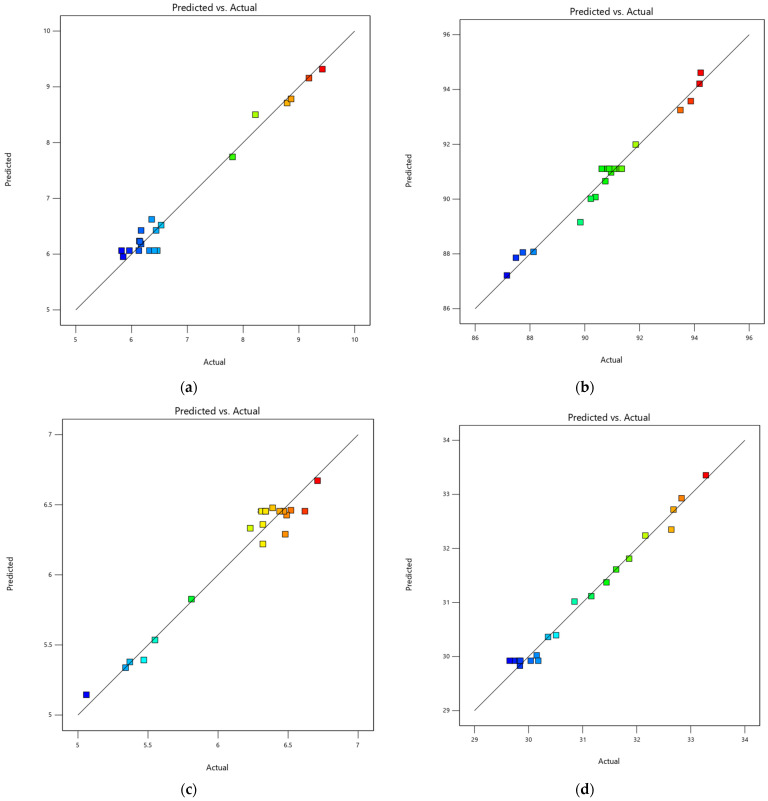
Plots comparing predicted and experimental data for responses including (**a**) moisture content, (**b**) whiteness index, (**c**) effective moisture diffusivity, and (**d**) activation energy.

The figures presented in Figure 5a–d demonstrate the comparison between experimental responses and expected responses. The presented figures demonstrate the degree of linearity observed in the data, revealing a strong correlation between the experimental and anticipated values. This relationship is visually illustrated in Figure 5a–d. The *R*^2^ values in Table 3 exhibit a range of 0.9651 to 0.9862, which suggests the statistical importance of the quadratic model. The table also indicates that all factors had a substantial impact on all replies, with a *p*-value less than 0.05. Hence, the updated models effectively encompass all relevant aspects [72]. The adj *R*^2^ values, which vary between 0.9337 and 0.9738, suggest that the concentration of sodium metabisulfite, citric acid concentration, and blanching time are all statistically significant factors in the drying process of cassava slices.

The coefficient of variation is a measure that represents the ratio of the standard deviation of the estimated values to the observed mean of the dependent variable. As stated by Fasuan and Akanbi [72], the *CV* serves as an indicator of the extent to which these models may be replicated and their correctness, with a stipulated criterion of *CV* < 10%. The study demonstrates a range of results spanning from 0.44% to 3.76%, indicating that the models utilized in this research exhibit a level of reproducibility. Hence, the models developed for the evaluated reactions in dehydrated cassava slices are capable of accurately characterizing the observed process dynamics. Equations (10)–(13) encompass the mathematical formulations that serve as models for the estimation of several parameters, including moisture content (*MC*), whiteness index (*WI*), activation energy (*E_a_*), and effective moisture diffusivity (*D_eff_*), pertaining to the dehydration process of cassava slices.

(10) $$\begin{array}{l} MC=6.06-0.2360\left(SM\right)-0.1940\left(CA\right)+1.1400\left(BT\right)+0.1255{\left(SM\right)}^{2}\\ \;+0.3655{\left(CA\right)}^{2}+1.3000{\left(BT\right)}^{2}+0.2450\left(SM\right)\left(CA\right)\\ \;+0.1775\left(SM\right)\left(BT\right)+0.1725(CA)(BT) \end{array}$$

(11) $$\begin{array}{l} WI=91.11-0.4500\left(SM\right)+0.3200\left(CA\right)-2.7300\left(BT\right)-0.5827{\left(SM\right)}^{2}\\ \;-0.7727{\left(CA\right)}^{2}+0.7773{\left(BT\right)}^{2}-0.1562\left(SM\right)\left(CA\right)\\ \;-0.1863\left(SM\right)\left(BT\right)-0.1537(CA)(BT) \end{array}$$

(12) $$\begin{array}{l} D_{eff}=6.45+0.1230\left(SM\right)+0.0940\left(CA\right)-0.4410\left(BT\right)+0.0950{\left(SM\right)}^{2}\\ \;-0.0700{\left(CA\right)}^{2}-0.6750{\left(BT\right)}^{2}-0.1013\left(SM\right)\left(CA\right)\\ \;-0.0288\left(SM\right)\left(BT\right)-0.0713(CA)(BT) \end{array}$$

(13) $$\begin{array}{l} E_{a}=29.92-0.2820\left(SM\right)-0.1700\left(CA\right)-0.6660\left(BT\right)+0.1905{\left(SM\right)}^{2}\\ \;+0.2705{\left(CA\right)}^{2}+1.7600{\left(BT\right)}^{2}-0.0138\left(SM\right)\left(CA\right)\\ \;+0.0488\left(SM\right)\left(BT\right)+0.0563(CA)(BT) \end{array}$$

where *SM* represents the sodium metabisulfite concentration (% *w*/*w*), *CA* represents the citric acid concentration (% *w*/*w*), and *BT* represents the blanching time (min).

A process of optimization was conducted in order to ascertain the most favorable pretreatment conditions for the production of cassava flour, with the objective of minimizing moisture content and activation energy values while maximizing whiteness index and effective moisture diffusivity values. The investigation into the optimization of the dependent and independent variables has demonstrated that the most favorable pretreatment conditions for the drying of cassava slices are achieved through the utilization of 1.31% citric acid, 1.03% sodium metabisulfite, and a blanching duration of 1.01 min. The convergence of optimal variables yielded a moisture content of 6.19% and a whiteness index of 92.00. The activation energy for the given process was determined to be 30.98 kJ/mol, while the effective moisture diffusivity was calculated to be 6.39 × 10^−9^ m^2^/s. Table 4 displays the results of a prediction and experiment that were carried out using optimized pretreatment parameters. The obtained response values suggest that the pretreatment procedure employed results in a mass transfer process that is reasonably energy-efficient and has enhanced characteristics.

### 3.3. Microstructure of Pretreated Cassava Flour

**Figure 6:**
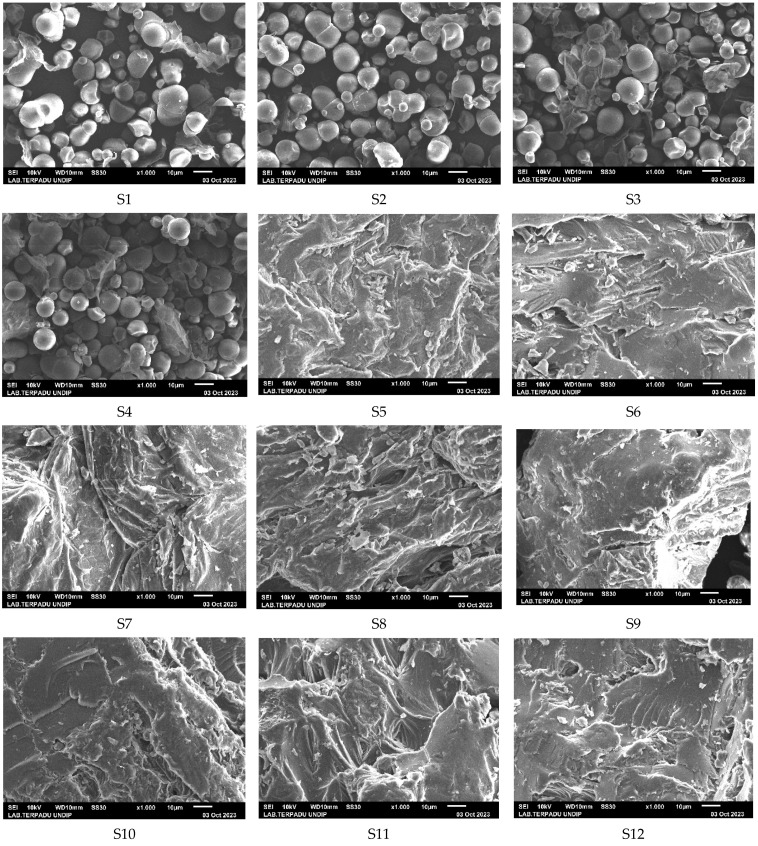

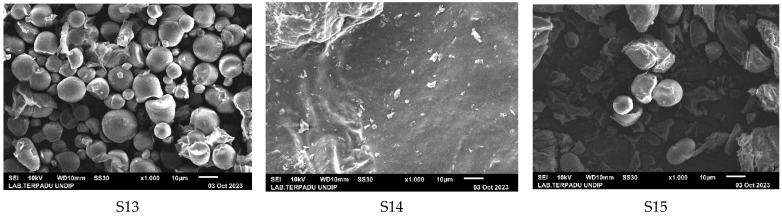
Microstructure of cassava flour with the pretreatment configuration described in Table 2 at 1000× magnification.

The microscopic findings of all samples, including both untreated and treated specimens, are depicted in Figure 6. Uniform oval and round granules were seen in the control sample (S1) as well as in the samples subjected to chemical treatment without blanching (S2, S3, S4, and S13). On the other hand, in samples subjected to a blanching duration of 4 min, the granules displayed a range of forms and sizes. The observed phenomenon can be attributed to variations in moisture content, with lower values indicating a more loosely arranged starch polymer structure, while higher values are indicative of a more densely packed molecular structure [73]. Tacer-Caba et al. [74] reported that the application of thermal processing techniques to food materials can result in an increased level of starch gelatinization. The presence of amylose in cassava starch has a significant impact on gelatinization and retrogradation [75].

Most of the blanched samples had granules that had gelatinized, which formed large aggregates with a block-like shape and rough, void-filled surfaces. However, sample S15, which underwent treatment with a solution containing 2% sodium metabisulfite and 2% citric acid and a 2-min blanching process, showed a distinct outcome. This result deviated from the observations made in the other blanched samples. The observed pretreatment configuration exhibited granule morphology that was characterized by oval and round shapes. According to the findings of the study by Kuttigounder et al. [76], partial starch gelatinization and retrogradation had a significant impact on the morphological characteristics of granules.

### 3.4. Dehydration Kinetics of the Optimized Pretreatment Parameters

Table 5 displays the numerical values of model constants and statistical parameters related to the process of cassava drying. The statistical parameters show that the *R*^2^ values range between 0.9816 and 0.9859, the *χ*^2^ values range between 0.0351 and 0.0564, the *RSME* values vary between 0.0015 and 0.0023, and the *SSE* values range between 0.0123 and 0.0203. According to these findings, the logarithmic model yielded the highest *R*^2^ value and the lowest *χ*^2^, *RSME*, and *SSE* values. Hence, this model is the best and most appropriate for characterizing the drying characteristics of cassava slices under the indicated ideal pretreatment conditions. According to Ajala et al. [65], the logarithmic model is the most suitable for modeling the drying process of cassava slices while utilizing a tunnel dryer.

Additional models, such as the Page model, have been employed to forecast the drying process of cassava slices using different treatments and drying techniques [76,77,78,79]. It is important to note that if different biomaterials are dried using the same pretreatment and drying configuration, it will be essential to analyze the models for each biomaterial individually in order to find the most suitable model.

## 4. Conclusions

Cassava, a tuberous plant, exhibits considerable potential for many applications in the realms of food, energy, and biofilm. The pretreatment process of cassava was modeled and optimized using a response surface methodology in order to provide efficient and cost-effective processing solutions for this plant. The results of the drying data optimization study indicate that the optimal pretreatment parameters for drying cassava slices are as follows: a citric acid concentration of 1.31%, a sodium metabisulfite concentration of 1.03%, and a blanching time of 1.01 min. The optimal conditions resulted in a moisture content of 6.19% and a whiteness index of 92.00, with an activation energy of 30.98 kJ/mol, and an effective moisture diffusivity of 6.39 × 10^−9^ m^2^/s. The suitability of the logarithmic model was demonstrated for cassava samples that were exposed to the ideal drying conditions. The logarithmic model yielded the highest *R*^2^ value and the lowest *χ*^2^, *RSME*, and *SSE* values. The results of microstructure analysis revealed that specific chemical and thermal pretreatment configurations (2% sodium metabisulfite, 2% citric acid, and blanching time for 2 min) produced particles in the form of round and oval granules. The thermal treatment of cassava tubers led to a greater extent of starch gelatinization in comparison to chemical processing. The findings disclosed in this study will have substantial implications for the advancement, evaluation, and analysis of dehydrating methodologies and systems designed for tubers.

## Figures and Tables

**Table 1:** Existing mathematical drying models.

Model’s Name	Model	Reference
Henderson and Pabis	$MR=a\;exp(-kt^{n})$	[44]
Page	$MR=exp(-kt^{n})$	[45]
Logarithmic	$MR=a\exp\left(-kt\right)+c$	[46]
Newton	$MR=\exp\left(-kt\right)$	[47]
Wang and Singh	$MR=1+at+bt^{2}$	[48]

**Table 2:** Design matrix and responses of dried cassava.

Sample	Uncoded and Coded Factors			Responses			
	*SM* (% *w*/*w*)	*CA* (% *w*/*w*)	*BT* (min)	*MC* (% db)	*WI*	*D_eff_* (m^2^/s) × 10^−9^	*E_a_* (kJ/mol)
S1	0 (−1)	0 (−1)	0 (−1)	7.81 ± 0.13	91.86 ± 0.06	5.81 ± 0.08	33.28 ± 0.43
S2	4 (+1)	0 (−1)	0 (−1)	6.44 ± 0.04	93.87 ± 0.05	6.23 ± 0.03	32.68 ± 0.38
S3	0 (−1)	4 (+1)	0 (−1)	6.53 ± 0.17	93.49 ± 0.07	6.32 ± 0.04	32.83 ± 0.56
S4	4 (+1)	4 (+1)	0 (−1)	6.17 ± 0.09	94.19 ± 0.03	6.52 ± 0.07	32.16 ± 0.44
S5	0 (−1)	0 (−1)	4 (+1)	9.42 ± 0.14	87.16 ± 0.08	5.06 ± 0.09	31.86 ± 0.25
S6	4 (+1)	0 (−1)	4 (+1)	8.79 ± 0.09	87.74 ± 0.05	5.55 ± 0.02	31.44 ± 0.39
S7	0 (−1)	4 (+1)	4 (+1)	8.86 ± 0.12	87.49 ± 0.02	5.47 ± 0.05	31.62 ± 0.47
S8	4 (+1)	4 (+1)	4 (+1)	9.18 ± 0.15	88.13 ± 0.08	5.37 ± 0.07	31.16 ± 0.52
S9	0 (−1)	2 (0)	2 (0)	6.17 ± 0.18	90.39 ± 0.04	6.49 ± 0.04	30.51 ± 0.24
S10	4 (+1)	2 (0)	2 (0)	5.85 ± 0.22	90.96 ± 0.03	6.71 ± 0.04	29.84 ± 0.35
S11	2 (0)	0 (−1)	2 (0)	6.36 ± 0.14	90.22 ± 0.06	6.48 ± 0.05	30.36 ± 0.27
S12	2 (0)	4 (+1)	2 (0)	6.14 ± 0.11	90.75 ± 0.07	6.39 ± 0.08	30.15 ± 0.43
S13	2 (0)	2 (0)	0 (0)	6.15 ± 0.06	94.23 ± 0.04	6.32 ± 0.06	32.64 ± 0.16
S14	2 (0)	2 (0)	4 (+1)	8.22 ± 0.08	89.84 ± 0.08	5.34 ± 0.03	30.85 ± 0.23
S15	2 (0)	2 (0)	2 (0)	6.46 ± 0.13	91.28 ± 0.03	6.31 ± 0.05	29.74 ± 0.22
S16	2 (0)	2 (0)	2 (0)	6.32 ± 0.09	90.83 ± 0.06	6.34 ± 0.08	29.83 ± 0.46
S17	2 (0)	2 (0)	2 (0)	6.13 ± 0.08	90.62 ± 0.09	6.47 ± 0.04	29.65 ± 0.27
S18	2 (0)	2 (0)	2 (0)	6.41 ± 0.05	91.07 ± 0.05	6.34 ± 0.03	30.04 ± 0.34
S19	2 (0)	2 (0)	2 (0)	5.96 ± 0.16	90.89 ± 0.02	6.44 ± 0.07	29.85 ± 0.18
S20	2 (0)	2 (0)	2 (0)	5.82 ± 0.12	91.35 ± 0.06	6.62 ± 0.05	30.18 ± 0.26

Note: values reported are means ± SD. *SM*, sodium metabisulfite concentrations; *CA*, citric acid concentrations; *BT*, blanching time; *MC*, moisture content; *WI*, whiteness index; *D_eff_*, effective moisture diffusivity; *E_a_*, activation energy.

**Table 3:** Analysis of variance (*F* value) for the response surface model of dried cassava.

Source	*Df*	Responses			
		*MC*	*WI*	*D_eff_*	*E_a_*
Model	9	45.6783 *	56.4101 *	30.7223 *	79.3221 *
*SM*	1	8.1370 *	12.5047 *	9.2542 *	21.1945 *
*CA*	1	5.4985 *	6.3233 *	5.4049 *	7.7023 *
*BT*	1	188.8693 *	459.5528 *	118.9614 *	118.2152 *
*SM*×*CA*	1	7.0156 *	1.2061 ^ns^	5.0166 *	0.0403 ^ns^
*SM*×*BT*	1	3.6824 ^ns^	1.7137 ^ns^	0.4045 ^ns^	0.5067 ^ns^
*BT*×*CA*	1	3.4778 ^ns^	1.1678 ^ns^	2.4842 ^ns^	0.6746 ^ns^
*SM*×*SM*	1	0.6323 ^ns^	5.7665 *	1.5181 ^ns^	2.6585 ^ns^
*CA*×*CA*	1	5.3659 *	10.1399 *	0.8242 ^ns^	5.3610 *
*BT*×*BT*	1	67.9459 *	10.2595 *	76.6424 *	227.1470 *
Lack of fit	5	1.07 ^ns^	3.14 ^ns^	1.41 ^ns^	0.9595 ^ns^
*R* ^2^		0.9763	0.9801	0.9651	0.9862
Adj *R*^2^		0.9549	0.9633	0.9337	0.9738
SD		0.2616	0.4024	0.1279	0.1937
Mean		6.9595	90.8180	6.1290	31.0335
*CV* (%)		3.7593	0.4431	2.0862	0.6242

Note: *, significant (*p* < 0.05); ^ns^, non-significant (*p* > 0.05); *SM*, sodium metabisulfite concentrations; *CA*, citric acid concentrations; *BT*, blanching time; *CV*, coefficient of variation; *R*^2^, determination coefficient; Adj *R*^2^, adjusted determination coefficient; *MC*, moisture content; *WI*, whiteness index; *D_eff_*, effective moisture diffusivity; *E_a_*, activation energy.

**Table 4:** Results of optimized pretreatment parameters for dried cassava.

Independent Variables	Optimum Level	Predicted				Experimental				*D*
		*MC* (% db)	*WI*	*D_eff_* (m^2^/s) × 10^−9^	*E_a_* (kJ/mol)	*MC* (% db)	*WI*	*D_eff_* (m^2^/s) × 10^−9^	*E_a_* (kJ/mol)	
*SM* (% *w*/*w*)	1.03	6.19	92.00	6.39	30.98	6.23 ± 0.12	92.18 ± 0.04	6.21 ± 0.03	30.64 ± 0.25	0.74
*CA* (% *w*/*w*)	1.31									
*BT* (min)	1.01									

Note: Values reported are means ± SD. *SM*, sodium metabisulfite concentrations; *CA*, citric acid concentrations; *BT*, blanching time; *D*, desirability.

**Table 5:** Model fitting result of optimized pretreatment parameters of dried cassava.

Model	Model Parameters	*R* ^2^	*χ* ^2^	*RMSE*	*SSE*
Henderson and Pabis	*a* = 0.9814, *k* = 0.2241	0.9823	0.0564	0.0018	0.0159
Page	*k* = 0.2318, *n* = 0.9914	0.9816	0.0556	0.0021	0.0164
Logarithmic	*a* = 1.0795, *k* = 0.1743, *c* = 0.1200	0.9859	0.0351	0.0015	0.0123
Newton	*k* = 0.2287	0.9816	0.0540	0.0016	0.0164
Wang and Singh	*a* = 0.1798, *b* = 0.0089	0.9821	0.0359	0.0023	0.0203

## Data Availability

Data are contained within the article.
